# Longitudinal prediction of outcome in idiopathic pulmonary fibrosis using automated CT analysis

**DOI:** 10.1183/13993003.02341-2018

**Published:** 2019-09-30

**Authors:** Joseph Jacob, Brian J. Bartholmai, Coline H.M. van Moorsel, Srinivasan Rajagopalan, Anand Devaraj, Hendrik W. van Es, Teng Moua, Frouke T. van Beek, Ryan Clay, Marcel Veltkamp, Maria Kokosi, Angelo de Lauretis, Eoin P. Judge, Teresa M. Jacob, Tobias Peikert, Ronald Karwoski, Fabien Maldonado, Elisabetta Renzoni, Toby M. Maher, Andre Altmann, Athol U. Wells

**Affiliations:** 1 Dept of Respiratory Medicine, University College London, London, UK; 2 Centre for Medical Image Computing, University College London, London, UK; 3 Dept of Radiology, Mayo Clinic Rochester, Rochester, MN, USA; 4 St Antonius ILD Center of Excellence, Dept of Pulmonology, St. Antonius Hospital, Nieuwegein, The Netherlands; 5 Division of Heart and Lungs, University Medical Center Utrecht, Utrecht, The Netherlands; 6 Dept of Radiology, Royal Brompton Hospital, London, UK; 7 Dept of Radiology, St. Antonius Hospital, Nieuwegein, The Netherlands; 8 Dept of Pulmonary Medicine, Mayo Clinic Rochester, Rochester, MN, USA; 9 Interstitial Lung Disease Unit, Royal Brompton Hospital, Royal Brompton and Harefield NHS Foundation Trust, London, UK; 10 Division of Pneumology, “Guido Salvini” Hospital, Garbagnate Milanese, Italy; 11 Dept of Respiratory Medicine, Aintree University Hospital, Liverpool, UK; 12 Dept of Radiology, St Georges Hospital, London, UK; 13 Dept of Physiology and Biomedical Engineering, Mayo Clinic Rochester, Rochester, MN, USA; 14 Division of Allergy, Pulmonary and Critical Care Medicine, Vanderbilt University Medical Center, Nashville, TN, USA; 15 NIHR Respiratory Clinical Research Facility, Royal Brompton Hospital, London, UK; 16 Fibrosis Research Group, National Heart and Lung Institute, Imperial College, London, UK

## Abstract

The advent of antifibrotic agents [1, 2] as standard of care in idiopathic pulmonary fibrosis (IPF) requires that new non-inferiority IPF drug trials will need to identify smaller declines of forced vital capacity (FVC). Marginal annualised FVC declines (between 5.00 and 9.99%) are particularly challenging to interpret as they might reflect measurement variation or genuine clinical deterioration [3]. Following on from previous baseline-only computed tomography (CT) analyses [4], the current study examined whether changes in computer features (CALIPER) across serial CT examinations could be considered as a trial co-endpoint, particularly with regard to adjudicating marginal FVC declines, and therefore improve the sensitivity of IPF drug trials.

## Abstract 2

**Change in the vessel-related structures, a computer-derived CT variable, is a strong predictor of outcome in idiopathic pulmonary fibrosis and can increase power in future drug trials when used as a co-endpoint alongside forced vital capacity change** http://bit.ly/2M7DfKS

To the Editor:

The advent of antifibrotic agents [1, 2] as standard of care in idiopathic pulmonary fibrosis (IPF) requires that new non-inferiority IPF drug trials will need to identify smaller declines of forced vital capacity (FVC). Marginal annualised FVC declines (between 5.00 and 9.99%) are particularly challenging to interpret as they might reflect measurement variation or genuine clinical deterioration [3]. Following on from previous baseline-only computed tomography (CT) analyses [4], the current study examined whether changes in computer features (CALIPER) across serial CT examinations could be considered as a trial co-endpoint, particularly with regard to adjudicating marginal FVC declines, and therefore improve the sensitivity of IPF drug trials.

Previous baseline IPF analyses identified that variable initiation time, dosages, durations and types of antifibrotic medication in study participants had a profound confounding effect on mortality relationships [4]. Consequently, analyses in the current manuscript were restricted to IPF patients not receiving anti-fibrotic therapy (discovery cohort: n=71 Royal Brompton Hospital patients presenting from January 2007 to December 2014); validation cohort: n=24 St Antonius Hospital, Nieuwegein patients presenting from January 2005 to June 2014 and n=23 Mayo Clinic Rochester patients presenting from January 2009 to June 2015). All patients had two non-contrast volumetric CT scans between 5 and 30 months apart (mean CT interval: discovery cohort 1.1 years; validation cohort 1.2 years) as part of their clinical care. Baseline diffusion capacity of the lung for carbon monoxide (*D*_LCO_) and FVC (baseline and longitudinal) were collected if performed within 3 months of the respective CTs. No patients were lost to follow-up.

Annualised FVC change was measured using a linear mixed effects model on all eligible timepoints to derive the best linear unbiased predictor (BLUP) as previously described using the lmer function from the R package lme4 [5]. A naïve estimate of FVC change was also examined using FVC measurements at the first and second CT timepoint. For the naïve estimate we computed annual relative change by dividing the absolute annual change by the baseline FVC value (relative). Dichotomised relative FVC declines (≥5% or ≥10%) were derived based on the naïve and BLUP estimates. Of the 27 CALIPER features examined [4], nine were measured on a whole lung level: total lung volume, normal parenchyma, vessel-related structures (CAL VRS), emphysema, honeycombing, reticular pattern and ground-glass opacity. Fibrosis extent summed reticular pattern and honeycombing. Interstitial lung disease extent additionally summed ground-glass opacification. 18 CAL VRS subdivisions were evaluated, separated according to lung zonal location: upper (UZ), middle (MZ) and lower zones (LZ), and cross-sectional area of structures in each zone: <5 mm^−2^, 5–10 mm^−2^, 10–15 mm^−2^, 15–20 mm^−2^, >20 mm^−2^. Volumes for all CALIPER features were converted into a percentage using CALIPER-derived total lung volume measurements [6, 7]. Absolute change in the derived 27 CT variables was annualised by dividing by the time interval between the two measurements (in years). Cox proportional hazards models examined CALIPER and FVC change variables in separate discovery and validation cohorts. Time was measured from the second CT. An event was either death (n=90) or transplantation (n=8). Each predictor variable was tested alone while correcting for patient age (at the second CT) and gender. Model fit was evaluated using the concordance index, which assesses how well the ordering of subjects for the actual time of the event agrees with the predicted time of the event. That is, for all subject pairs it checks whether the subject who had the event first was also the subject predicted to have the event first. A C-index of 0.5 indicates random performance, where in 50% of cases the subject with the earlier event was predicted to be the subject with the later event. Approval for this study of clinically indicated CT and pulmonary function data was obtained from Liverpool Research Ethics Committee (reference: 14/NW/0028) and the Institutional Ethics Committee of the Royal Brompton Hospital, Mayo Clinic Rochester and St Antonius Hospital, Nieuwegein. Informed patient consent was not required.

Our study findings identified absolute CAL VRS and UZ VRS increases as the strongest survival predictors in discovery and validation cohorts (figure 1a). Both variables were at least equivalent to FVC change when evaluated using C-indices. Significant but weak correlations (r=−0.42, p=1.8×10^−6^) were identified between FVC change and absolute VRS change (Pearson's correlation).

**FIGURE 1:**
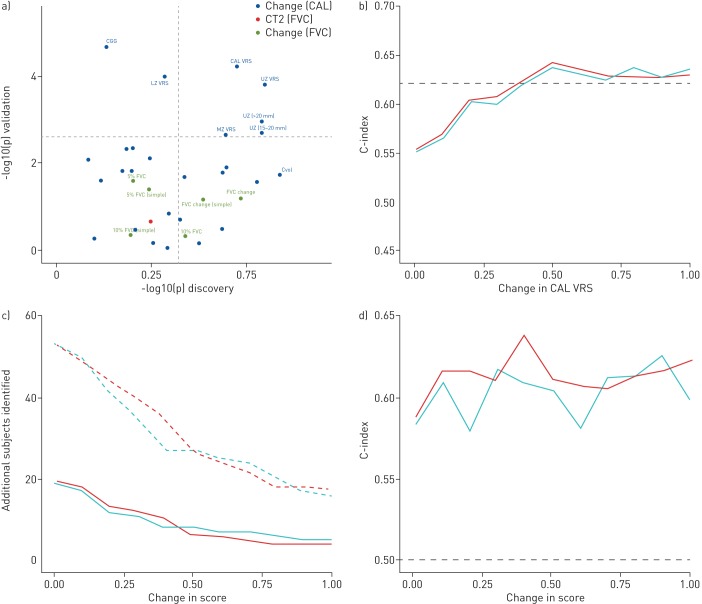
a) Scatterplot of -log10 p-values for various computer-derived (CALIPER) variables (blue points) and forced vital capacity (FVC) decline (green points) in patients not exposed to antifibrotic medication in the discovery cohort (*x*-axis, n=71) and validation cohort (*y*-axis, n=47). Horizontal and vertical dotted lines represent the Li and Ji corrected cut-off for statistical significance. FVC decline was calculated using two methods: naïve estimate from two timepoints aligned with the two computed tomography (CT) timepoints (simple) and using best linear unbiased predictions. FVC change was expressed as a continuous variable (FVC change), and at ≥5% decline and ≥10% decline thresholds. The FVC value at the timepoint of the second CT scan (red dot) was used to benchmark expressions of FVC decline. The pulmonary vessel-related structure score (CAL VRS) was subdivided according to zonal location (UZ VRS: upper zone; MZ VRS: middle zone; LZ VRS: lower zone) and structure cross-sectional area in each zone (<5 mm^−2^, 5–10 mm^−2^, 10–15 mm^−2^, 15–20 mm^−2^, >20 mm^−2^). b) C-indices (a measure of goodness of fit of a model) for models examining thresholds of change in CAL VRS examined against a 10% FVC decline threshold. The horizontal dotted black line indicates the C-index for a 10% FVC decline threshold model examining the relevant FVC threshold alone. The blue line demonstrates the C-indices for models when a CAL VRS threshold alone was examined. The red line demonstrates the C-indices for models where a binary variable indicated a “joint endpoint”, *i.e.* either the CALIPER or FVC threshold had been reached. c) Additional patients that would reach an endpoint (*y*-axis), if CAL VRS (red) or upper-zone vessel related structure (UZ VRS, blue) thresholds of change (*x*-axis) were examined in addition to FVC decline thresholds. The FVC decline thresholds examined included a ≥5% FVC decline threshold (solid line) and a ≥10% FVC decline threshold (dotted line). d) C-indices (*y*-axis) for models containing varying thresholds (*x*-axis) of CAL VRS (red) or UZ VRS (blue) in patients with an FVC between 5% and 10%. The horizontal dashed black line indicates the C-index 0.5, *i.e.* random performance.

Both study populations were then combined and Cox proportional hazards models examined thresholds of CAL VRS and UZ VRS change measured against relative FVC decline thresholds of ≥5% and ≥10% adjusted for patient age and gender. The predictive performance of FVC-based indicator variables was compared to VRS indicator variables, either used alone or when combined with an FVC-based indicator variable as a “joint endpoint”. The joint endpoint reflected whether the FVC decline or the VRS increase was achieved with estimates based on 500 bootstrap replicates (n=118). We estimated the number of additional patients that would reach either a ≥5% or ≥10% predicted FVC threshold or a preselected CAL VRS/UZ VRS change threshold in a drug trial setting. Further, we computed the Kaplan–Meier estimator for different subgroups of patients (n=118) depending on whether they reached the FVC or VRS threshold or both (using the SPSS Kaplan–Meier function [8]). Finally, we examined mortality prediction in patients with a BLUP estimated relative decline in FVC of >5% but <10% not receiving antifibrotics (n=41).

In the combined study population, multivariate Cox mortality models demonstrated that a CAL VRS increase of ≥0.30% independently predicted mortality when evaluated against a ≥10% FVC decline threshold. When CAL VRS increased by ≥0.50%, a ≥10% FVC decline threshold no longer significantly contributed to mortality prediction. At CAL VRS (figure 1b) and UZ VRS increases of ≥0.40%, no difference in model C-index was seen when compared to a ≥10% FVC decline threshold. The C-index was unchanged when using either a solitary CALIPER endpoint (CAL VRS or UZ VRS increase), or a combined endpoint of an increase in a CALIPER variable and an FVC ≥10% decline threshold. Results were maintained when CALIPER variable change thresholds were compared to a ≥5% FVC decline threshold.

79/118 (67%) patients reached a CAL VRS of ≥0.40% change whilst 54/118 (46%) reached a ≥10% FVC decline threshold (p=0.0003). 89/118 (75%) patients reached either the CAL VRS threshold of ≥0.40% change or ≥10% FVC decline threshold (figure 1c). Use of a CAL VRS increase threshold of ≥0.40% change identified 35/118 (30%) more patients reaching an endpoint than the ≥10% FVC decline threshold alone. Similarly, at least 30% more patients reached an endpoint when an UZ VRS threshold was used alongside a ≥10% FVC decline threshold (figure 1c). When CAL VRS and UZ VRS elevation thresholds were examined against a ≥5% FVC decline threshold, additional patients reaching an endpoint were again identified. When all patients with an FVC decline more than 5% and less than 10% were subanalysed, CAL VRS thresholds ≥0.40% change demonstrated C-indices that were at least equivalent to a ≥10% FVC decline threshold (figure 1d).

Our findings demonstrate that in independent discovery and validation populations, an absolute increase in a computer-derived variable, the vessel-related structures (CAL VRS), strongly predicts mortality in IPF patients not exposed to antifibrotic medication. Patients exhibiting a CAL VRS increase ≥0.40% were different to those experiencing an FVC decline ≥10%. Accordingly, if a composite endpoint of CAL VRS ≥0.40% increase and/or ≥10% FVC decline were used in a drug trial setting, 30% more patients would reach the composite endpoint than a solitary endpoint of ≥10% FVC decline. Our findings also suggest the utility of a CAL VRS threshold ≥0.40% increase as an arbitration tool for marginal FVC declines (between 5.0 and 9.9%).

The weak correlations between FVC change and VRS change indicate that both variables represent important yet distinct surrogate measures of mortality and argues for their integration as co-endpoints rather than selecting one over another. A ≥0.40% increase in VRS across a cohort appeared to be the most accurate measure of change in VRS, when considering both its prognostic effect when judged against FVC decline and its sensitivity as an endpoint. In an individual, whilst the most accurate threshold for VRS change may also be a ≥0.40% threshold, further work is necessary to establish optimal thresholds for use in clinical practice, as just having knowledge of the range of change of a variable does not of course provide any statement of the clinical significance of that change. For example, it was noticeable that more extreme VRS cut-offs, *e.g.* 0.75%, made even more of a difference in model fit and C-index than a ≥0·40% threshold. But we cannot know how often such a magnitude of VRS change would be seen in a clinical trial population. A logical next analytic step would therefore be to evaluate VRS change in a well-controlled drug trial population receiving antifibrotics at a standardised dosing regimen.

The validity of VRS change was considered according to the OMERACT filter criteria for IPF clinical trial domains [9]. Regarding truth and discrimination criteria, VRS change was considered to be more discriminatory than FVC change at predicting outcome, with potential for use as a continuous variable (with no loss of signal strength), or as a binary threshold alongside an FVC decline threshold to improve endpoint sensitivity. The variable therefore satisfies construct, content and criterion validity and demonstrates sensitivity to change.

The specific impact on VRS change of differing inspiratory effort, acquisition or reconstruction parameters has not been systematically investigated, and further study is indicated. However, our analysis of this measure in a heterogeneous dataset from multiple institutions suggests this is robust. CALIPER outputs are eminently interpretable and feasible to perform but real-world utility of VRS for clinical trials relies on availability of repeated CTs and the computer algorithm, and is therefore limited when compared to FVC measurements.

There were limitations to the current study. Though there were similar average CT intervals between the two study cohorts and change in CT variables were reported as annualised change, the CTs time intervals were not standardised in this retrospective analysis. This lack of standardisation reflects real world clinical practice but may have biased our findings in patients with shorter or longer CT follow up intervals. Whilst the ideal study would have rigorous protocol-led control of serial CT and functional measurements and antifibrotic use, no such study yet exists and were it to begin today, outcome data may only be available several years hence. Accordingly, we believe our analyses capture a realistic contemporary cross-section of IPF data points.

In conclusion, we have demonstrated for the first time that change in a computer-derived variable, vessel-related structures, which has no visual correlate is a powerful surrogate for mortality in IPF. VRS change correlates weakly with FVC change and identifies different poor-outcome patients than a ≥10% FVC decline threshold. Use of a VRS threshold of ≥0.40% change alongside a ≥10% FVC decline threshold can identify 30% more patients that reach an endpoint and argues for the consideration of VRS change as an IPF drug trial co-endpoint to adjudicate indeterminate FVC declines of 5.0–9.9%.

## Shareable PDF

10.1183/13993003.02341-2018.Shareable1 This one-page PDF can be shared freely online.
Shareable PDF ERJ-02341-2018.Shareable
