# Supplementary material for: Longitudinal prediction of outcome in idiopathic pulmonary fibrosis using automated CT analysis
Source: Eur Respir J. 2019 Sep 30;54(3):1802341. doi: 10.1183/13993003.02341-2018 (PMC6860992; doi:10.1183/13993003.02341-2018)
Supplement: Supplementary file 1 [file ERJ-02341-2018.Shareable.pdf]

Longitudinal prediction of outcome in idiopathic pulmonary fibrosis using automated CT analysis

Joseph Jacob [1,2], Brian J. Bartholmai[3], Coline H.M. van Moorsel[4,5], Srinivasan Rajagopalan[3], Anand Devaraj[6], Hendrik W. van Es[7], Teng Moua[8], Frouke T. van Beek[4], Ryan Clay[8], Marcel Veltkamp[4,5], Maria Kokosi[9], Angelo de Lauretis[10], Eoin P. Judge[11], Teresa M. Jacob[12], Tobias Peikert[8], Ronald Karwoski[13], Fabien Maldonado[14], Elisabetta Renzoni[9], Toby M. Maher[15,16], Andre Altmann [2] and Athol U. Wells[9]

Affiliations: [1]Dept of Respiratory Medicine, University College London, London, UK. [2]Centre for Medical Image Computing, University College London, London, UK. [3]Dept of Radiology, Mayo Clinic Rochester, Rochester, MN, USA. [4]St Antonius ILD Center of Excellence, Dept of Pulmonology, St. Antonius Hospital, Nieuwegein, The Netherlands. [5]Division of Heart and Lungs, University Medical Center Utrecht, Utrecht, The Netherlands. [6]Dept of Radiology, Royal Brompton Hospital, London, UK. [7]Dept of Radiology, St. Antonius Hospital, Nieuwegein, The Netherlands. [8]Dept of Pulmonary Medicine, Mayo Clinic Rochester, Rochester, MN, USA. [9]Interstitial Lung Disease Unit, Royal Brompton Hospital, Royal Brompton and Harefield NHS Foundation Trust, London, UK. [10]Division of Pneumology, "Guido Salvini" Hospital, Garbagnate Milanese, Italy. [11]Dept of Respiratory Medicine, Aintree University Hospital, Liverpool, UK. [12]Dept of Radiology, St Georges Hospital, London, UK. [13]Dept of Physiology and Biomedical Engineering, Mayo Clinic Rochester, Rochester, MN, USA. [14]Division of Allergy, Pulmonary and Critical Care Medicine, Vanderbilt University Medical Center, Nashville, TN, USA. [15]NIHR Respiratory Clinical Research Facility, Royal Brompton Hospital, London, UK. [16]Fibrosis Research Group, National Heart and Lung Institute, Imperial College, London, UK.

Correspondence: Joseph Jacob, Dept of Respiratory Medicine, Rayne Institute, University Street, University College London, WC1E 6JF, UK. E-mail: j.jacob@ucl.ac.uk

@ERSpublications
Change in the vessel-related structures, a computer-derived CT variable, is a strong predictor of outcome in idiopathic pulmonary fibrosis and can increase power in future drug trials when used as a co-endpoint alongside forced vital capacity change http://bit.ly/2M7DfKS

Cite this article as: Jacob J, Bartholmai BJ, van Moorsel CHM, *et al.* Longitudinal prediction of outcome in idiopathic pulmonary fibrosis using automated CT analysis. *Eur Respir J* 2019; 54: 1802341 [https://doi.org/10.1183/13993003.02341-2018].

This single-page version can be shared freely online.

To the Editor:

The advent of antifibrotic agents [1, 2] as standard of care in idiopathic pulmonary fibrosis (IPF) requires that new non-inferiority IPF drug trials will need to identify smaller declines of forced vital capacity (FVC). Marginal annualised FVC declines (between 5.00 and 9.99%) are particularly challenging to interpret as they might reflect measurement variation or genuine clinical deterioration [3]. Following on from previous baseline-only computed tomography (CT) analyses [4], the current study examined whether changes in computer features (CALIPER) across serial CT examinations could be considered as a trial co-endpoint, particularly with regard to adjudicating marginal FVC declines, and therefore improve the sensitivity of IPF drug trials.

Copyright ©ERS 2019 This version is distributed under the terms of the Creative Commons Attribution Licence 4.0.
